# Northward range expansion of *Ixodes scapularis* evident over a short timescale in Ontario, Canada

**DOI:** 10.1371/journal.pone.0189393

**Published:** 2017-12-27

**Authors:** Katie M. Clow, Patrick A. Leighton, Nicholas H. Ogden, L. Robbin Lindsay, Pascal Michel, David L. Pearl, Claire M. Jardine

**Affiliations:** 1 Department of Pathobiology, Ontario Veterinary College, University of Guelph, Guelph, Ontario, Canada; 2 Department of Pathology and Microbiology, Faculty of Veterinary Medicine, University of Montréal, Saint-Hyacinthe, Quebec, Canada; 3 National Microbiology Laboratory, Public Health Agency of Canada, Saint-Hyacinthe, Quebec, Canada; 4 National Microbiology Laboratory, Public Health Agency of Canada, Winnipeg, Manitoba, Canada; 5 Office of the Chief Science Officer, Public Health Agency of Canada, Ottawa, Ontario, Canada; 6 Department of Population Medicine, Ontario Veterinary College, University of Guelph, Guelph, Ontario, Canada; 7 Canadian Wildlife Health Cooperative, Ontario Veterinary College, University of Guelph, Guelph, Ontario, Canada; University of Toledo College of Medicine and Life Sciences, UNITED STATES

## Abstract

The invasion of the blacklegged tick, *Ixodes scapularis* into Ontario, Canada poses a significant risk to public health because it is a vector for numerous pathogens, including *Borrelia burgdorferi* sensu stricto, the causative agent of Lyme disease. Baseline field sampling in 2014 and 2015 detected *I*. *scapularis* and *B*. *burgdorferi* at sites across southern, eastern and central Ontario, including a hot spot in eastern Ontario. A “speed of spread” model for *I*. *scapularis* developed by Leighton and colleagues (2012) estimated that the tick’s range was expanding northward at 46 km/year. In 2016, we revisited a subset of sites sampled in 2014 and 2015 to understand the changing nature of risk, and assess whether the rate of tick invasion is consistent with the speed of spread estimate. Ticks were collected via tick dragging at 17 out of 36 sites, 5 of which were new sites for *I*. *scapularis*. Samples were positive for *B*. *burgdorferi* at 8 sites. No other *I*. *scapularis*-borne pathogens were detected. Centrographic statistics revealed an increase in the dispersion of *I*. *scapularis* positive sites in eastern Ontario. Field data for each site were then compared to the model’s predicted year of establishment for each census subdivision. Our findings illustrate that the range expansion of *I*. *scapularis* and the emergence of *B*. *burgdorferi* is ongoing, and provide short timescale evidence of the processes associated with *I*. *scapularis* spread. The range front appears to be moving at a rate of ~46 km/year, with colonization of the tick behind this range front occurring at a slower and heterogeneous rate. Assessment of site-level ecological factors did not provide any insight into the underlying processes that may be influencing the colonization of *I*. *scapularis* in specific areas. Ongoing field sampling is needed to monitor this dynamic process. This study highlights the current geographic risk associated with Lyme disease, which can be used to target public health interventions to the areas of greatest risk.

## Introduction

The blacklegged tick, *Ixodes scapularis*, has undergone extensive range expansion in the recent past. Reforestation of large areas of the United States created suitable habitat for the tick and the primary host for the adult life stage, the white-tailed deer, *Odocoileus virginianus* [1]. *Ixodes scapularis* re-emerged from two foci: one in the northeastern United States, and the other in the Midwest [2,3]. This tick is a vector for numerous pathogens of public health significance, including *Borrelia burgdorferi* sensu stricto (herein referred to as *B*. *burgdorferi*), the causative agent of Lyme disease in North America [4]. The northern extent of the range of *I*. *scapularis* is currently expanding into Canada [5,6].

Invasion of *I*. *scapularis* into Ontario, Canada involves several dynamic processes. Since 1990, the number of established, reproducing populations of *I*. *scapularis* has dramatically increased, with a subsequent increase in the incidence of human Lyme disease cases [6–8]. Each year, migratory birds likely introduce millions of ticks from the northeastern United States into Canada [9]. This long-distance (≤425 km) dispersal of ticks seeds new areas and may lead to the establishment of new reproducing populations [9]. With climate change, this process is anticipated to continue, as more northern areas become climatically suitable for the tick [7,10,11].

At a finer scale, there is limited knowledge of the mechanisms of tick dispersal. Ticks only move a few metres on their own during each life stage [12]. Therefore, local range expansion is dependent on the movement of hosts, including white-tailed deer, small mammals and ground-dwelling birds [13].

Significant effort has been placed into predicting future expansion of *I*. *scapularis* and the risk of *B*. *burgdorferi* in the province. Leighton and colleagues [14] used 19 years of passive surveillance data to assess the factors determining the northward speed of spread, and then provided an estimate of the speed of spread of the northern range front. The range front was predicted to continue expanding northward at approximately 46 km/year, with expected variation in speed between 35 to 55 km/year depending on temperature conditions. The accompanying colonization or ‘filling in’ of suitable woodland areas behind this range front by *I*. *scapularis* would occur rapidly afterwards. Stochastic fade-out of early populations can also happen during this time [15]. In this region, the spread of populations of the tick has, in general, been followed by the spread of the bacteria that the tick transmits. In eastern Canada, including Ontario, estimates to date have suggested that there is a five-year lag between the establishment of a reproducing population of *I*. *scapularis* and a detectable, sustained cycle of transmission of *B*. *burgdorferi* [16].

In 2014, we conducted large-scale field sampling for *I*. *scapularis* across southern, eastern and central Ontario to determine the distribution of the tick and the risk of Lyme disease [8]. *Ixodes scapularis* was collected across the study area, with a hot spot of the tick detected in eastern Ontario. Additional field sampling was conducted in 2015 [17]. Over the two years of sampling, the tick was detected at 29 of 154 sites (18.8%) [8,17].

With knowledge of the baseline distribution of *I*. *scapularis* and *B*. *burgdorferi* in Ontario, we are now able to obtain a snapshot of the processes associated with spread. If the range front of *I*. *scapularis* is spreading northward at a constant 46 km/year, as estimated by Leighton and colleagues [14], then the tick should be newly detected at sites within a 46-km radius of areas with a high prevalence of sites with *I*. *scapularis* in 2014 or 2015. If an *I*. *scapularis* population establishes free of *B*. *burgdorferi* [16], then *I*. *scapularis* at newly detected sites should be free of *B*. *burgdorferi*, and a subset of sites with the presence of *I*. *scapularis* in 2014 or 2015 may have ticks positive for *B*. *burgdorferi* in 2016.

In this study, we conducted field sampling for *I*. *scapularis* at a subset of previously visited sites in Ontario. The objectives were to re-examine the distribution of *I*. *scapularis* and *B*. *burgdorferi* within a zone of emergence and apply these field data to assess whether short timescale changes in tick populations are consistent with the speed of spread estimated by Leighton et al. [14].

## Methods

### Site selection

Assessment of the speed of spread of the range front requires knowledge on the current geographic limits of *I*. *scapularis* in the province. Since it was not feasible to conduct field sampling at every possible site, a proxy measure of the current range was required. The primary cluster detected in 2014 contained 15 of the 21 sites with *I*. *scapularis* and was deemed to be suitable baseline measurement from which to assess *I*. *scapularis* spread.

**Fig 1:**
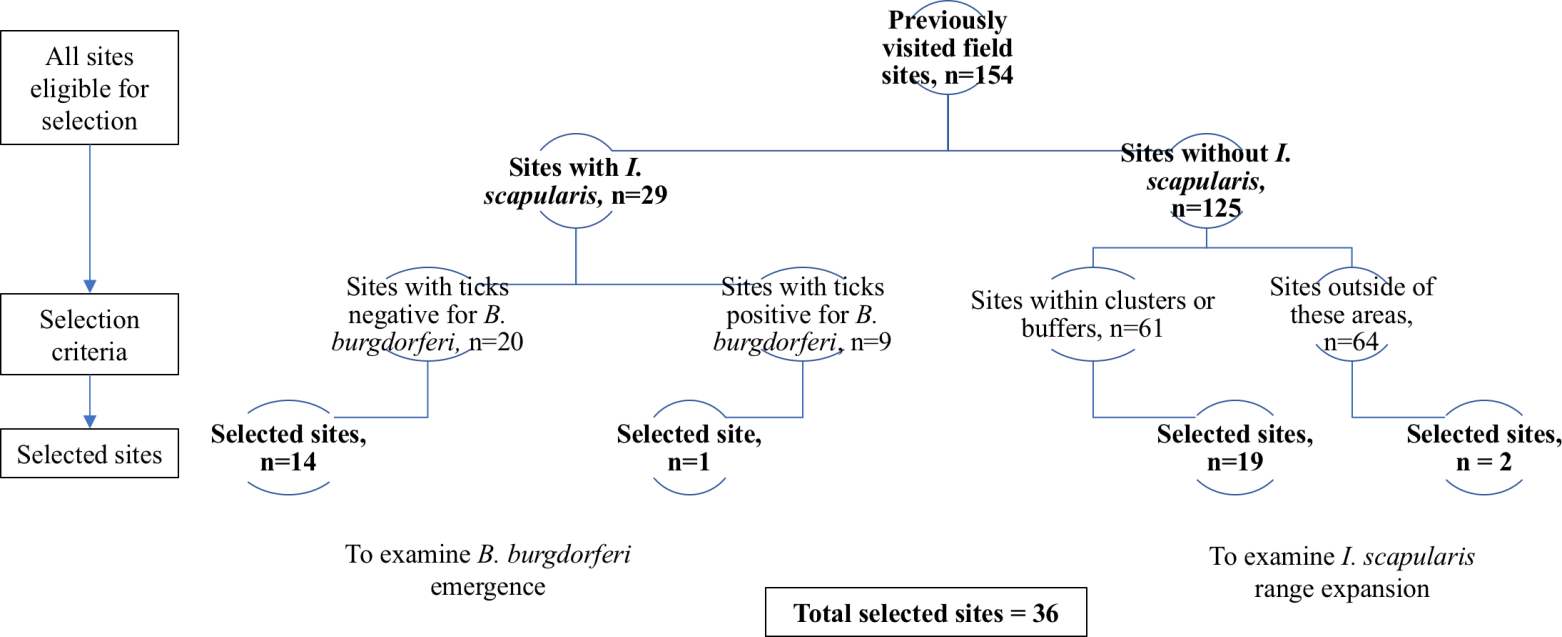
Algorithm for site selection. Sites were selected from the 154 sites visited in 2014 or 2015 [8,17]. Sites were classified based on *I*. *scapularis* status. If *I*. *scapularis* had not been previously detected at the site, the sites were further classified based on the results of the spatial scan statistics and creation of buffers. Sites within this category were selected to explore the range expansion of *I*. *scapularis*. If *I*. *scapularis* had previously been detected at the site, the site was further classified by *B*. *burgdorferi* status. Sites within this category were selected to explore the emergence of *B*. *burgdorferi*.

Site selection and initial cluster analysis were previously described [8]. Cluster analysis was updated to include additional field data from 2015. A spatial scan statistic using a Bernoulli probability model was applied to retrospectively identify areas (i.e., clusters) of high prevalence of *I*. *scapularis*. No geographical overlap of clusters was permitted, with the maximum size of a cluster set at 50% or less of the total population. Statistical significance was assessed based on 999 Monte Carlo replications. Sites that were positive for *I*. *scapularis* either in 2014 or 2015 were included as cases, as well as other sites already known to have established populations of *I*. *scapularis* based on previous surveillance studies in the region [18]. Any site where *I*. *scapularis* was not detected was included as a control. Analysis was conducted using SaTScan v9.4.4 (www.satscan.org, 2016) with a significance level of α = 0.05. The site locations and the results of cluster analysis were mapped using ArcGIS 10.5 (Esri, Redlands, CA; 2016). The Universal Transverse Mercator (UTM) North American Datum (NAD) 1983 projection (zones 18N and 17N) was chosen to accurately calculate distance [19]. Both 46-km and 92-km buffers were created around the perimeters of the primary and secondary spatial clusters to represent potential spatial spread of *I*. *scapularis* over one or two years, respectively, according to the estimate by Leighton et al. [14]. An algorithm for site selection was then applied (Fig 1). Any previously visited field site that was negative for *I*. *scapularis* and either within the clusters, or the buffer zones was eligible for selection for analysis of *I*. *scapularis* spread. Any site within the entire study area that was previously positive for *I*. *scapularis*, but negative for *B*. *burgdorferi* was eligible for analysis for the invasion of *B*. *burgdorferi*. Final selection from these categories of eligible sites was conducted using convenience sampling (e.g., permission to access site). In total, 36 sites were selected. Specifically, 21 sites were selected to assess spatial spread of *I*. *scapularis*; 1 site was within the primary cluster, 9 sites were within the 46-km buffer of the primary cluster, 5 sites were within the 92-km buffer of the primary cluster, 4 sites were within the 92-km buffer of the secondary cluster, and 2 sites were outside of both buffer regions. To assess *B*. *burgdorferi* invasion, 15 sites were selected.

### Field sampling

Thirty-six sites were visited once during either the spring (May and June) or fall (September and October) of 2016. Tick dragging was conducted by dragging a 1 m^2^ white flannel drag cloth across the forest floor and surrounding vegetation for a total of 3 person-hours. The drag cloth was checked for ticks every 3 minutes, during which time the timer was stopped. All life stages of *I*. *scapularis* were collected and stored in 70% ethanol for species verification and laboratory testing.

### Ecological data

Site aspect, tree coverage, understory density and type, soil composition and moisture, and depth of litter layer were previously recorded for each site [17]. Multiple measurements were taken for each site for previous analysis, so data were aggregated for each variable to the site-level for this study.

### Laboratory testing

All adult, nymphal and if required, larval *I*. *scapularis* were sent to the National Microbiology Laboratory (NML) at Winnipeg (Public Health Agency of Canada, Winnipeg, Manitoba, Canada) for species verification. Adult and nymphal *I*. *scapularis* subsequently underwent testing at NML for *B*. *burgdorferi*, and four other *I*. *scapularis*-borne pathogens: *Anaplasma phagocytophilum*, *B*. *miyamotoi*, *Babesia microti* and Powassan Virus.

Laboratory analyses have been previously described [20]. Briefly, DNA was extracted using DNeasy 96 tissue kits (QIAGEN Inc., Mississauga, Canada). Initial screening for *Borrelia* spp. was conducted using the 23s ribosomal RNA real-time polymerase chain reaction (PCR) assay. This was coupled with the msp2 real-time PCR for the detection of *A*. *phagocytophilum* [21]. Samples that tested positive for *Borrelia* spp. were subjected to the ospA real-time PCR to detect *B*. *burgdorferi* and the IGS real-time PCR for *B*. *miyamotoi*. *Borrelia miyamotoi*-positive samples were then verified with the glpQ real-time PCR [22]. Real-time PCR for the CCTeta gene was used to detect *B*. *microti* [23]. To ensure contamination did not occur during extraction and PCR runs, water blanks were used.

### Model exploration

The original output of the parametric survival regression developed by Leighton and colleagues [14] to determine the speed of spread of *I*. *scapularis* in Canada was used for model exploration. In building this model, 19 years of passive surveillance data (1990–2008) were incorporated with data on short and long distance tick dispersal and ecological factors of annual cumulative degree days above 0°C (DD>0°C), average annual rainfall, elevation, and percent deciduous or mixed deciduous forest, all at the level of census subdivision (CSD). The final model included DD>0°C, elevation, annual rainfall and both short and long distance dispersal, and estimated range expansion at 46 km/year northward, with variation between 35 km to 55 km, depending on if DD>0°C were cooler or warmer than the period from 1990–2008, respectively [14]. The model output included the number of years to establishment from 1991 (number from 0 to n), and the predicted year of *I*. *scapularis* establishment with standard error (year from 1991 to n) for each census subdivision (CSD) in our study area. The predicted year of establishment was compared to the site status from each year of field sampling to determine if the time of detection of *I*. *scapularis* aligned with the model output. The standard error was used to calculate the 95% confidence interval for each prediction, and findings of field sampling were also compared to the range of dates. All year predictions were rounded down to the nearest whole number.

Sites were classified as early-to-establish if *I*. *scapularis* was detected prior to the predicted date for the CSD, on-time if the year *I*. *scapularis* was detected aligned with the model prediction, or late-to-establish if *I*. *scapularis* was not detected by the predicted year of establishment. If a site was negative for *I*. *scapularis* with a future predicted year of establishment, the site was labelled as pre-emergence. Similarly, if a site was positive for *I*. *scapularis* with a predicted year of establishment in advance of our sampling time frame, the site was labelled as post-emergence.

To verify if the estimated rate of range expansion could be slower (35 km/year) or faster (55 km/year) based on DD>0°C, the average DD>0°C for each CSD was calculated for the time frame directly preceding our study (2009–2013) and compared to the average DD>0°C for 1990–2008 time frame. Climatic data was accessed from Environment Canada and calculated as described in Leighton et al. [14].

### Statistical analysis

Prevalence and exact 95% confidence intervals (CI) were calculated for sites with *I*. *scapularis* and sites with *B*. *burgdorferi*.

Multinomial logistic regression was used to assess the relationship between the number of years to establishment from the speed of spread model (explanatory variable) and the site status for *I*. *scapularis* (established, risk area, negative) (i.e., is there a statistically significant relationship between the *I*. *scapularis* site status and the predicted number of years to establishment?). Sites were classified as established if *I*. *scapularis* was detected both years, and a risk area if *I*. *scapularis* was detected only in the second year. To test the assumption of linearity, ordinary (binary) logistic regression models were generated for each outcome level. We considered the assumption of linearity to be met if the lowess curve formed a straight line against the log odds of the outcome. Model fit was assessed using the Fagerland, Hosmer, and Bofin goodness of fit test [24]. The null hypothesis that the model fits was rejected if the goodness-of-fit test was significant (p<0.05).

To determine if ecological factors had an influence on the speed of spread of *I*. *scapularis*, univariable analysis with logistic regression was conducted. The outcome was the site status late-to-establish (versus all other statuses), and the explanatory variables were the site-level ecological, including the difference of DD>0°C between the time frame preceding the study (2009–2013) and the time frame of model development (1990–2008). If the number of observations per category was less than five, exact logistic regression was conducted [25].

All statistical analyses were conducted using STATA version 14.0 (STATACorp, College Station, TX; 2016) with a significance level of α = 0.05.

### Spatial analysis

Centrographic statistics of mean centre and standard deviational ellipse were calculated for both the baseline field sampling (2014–2015) and follow-up field sampling (2016) time frames in the area around the primary cluster. The standard deviational ellipse was chosen as it provides a measure dispersion of sites with *I*. *scapularis*, and can be used to assess if dispersion changes over time if multiple time points of data are available [26]. Sites that were positive for *I*. *scapularis* during that field sampling time frame were used as point locations. Analysis was conducted using CrimeStat v3.3 [27], and results were projected using ArcGIS (Esri, Redlands, CA; 2016). It was not possible to conduct these calculations for the area around the secondary cluster as there were not sufficient data.

**Fig 2:**
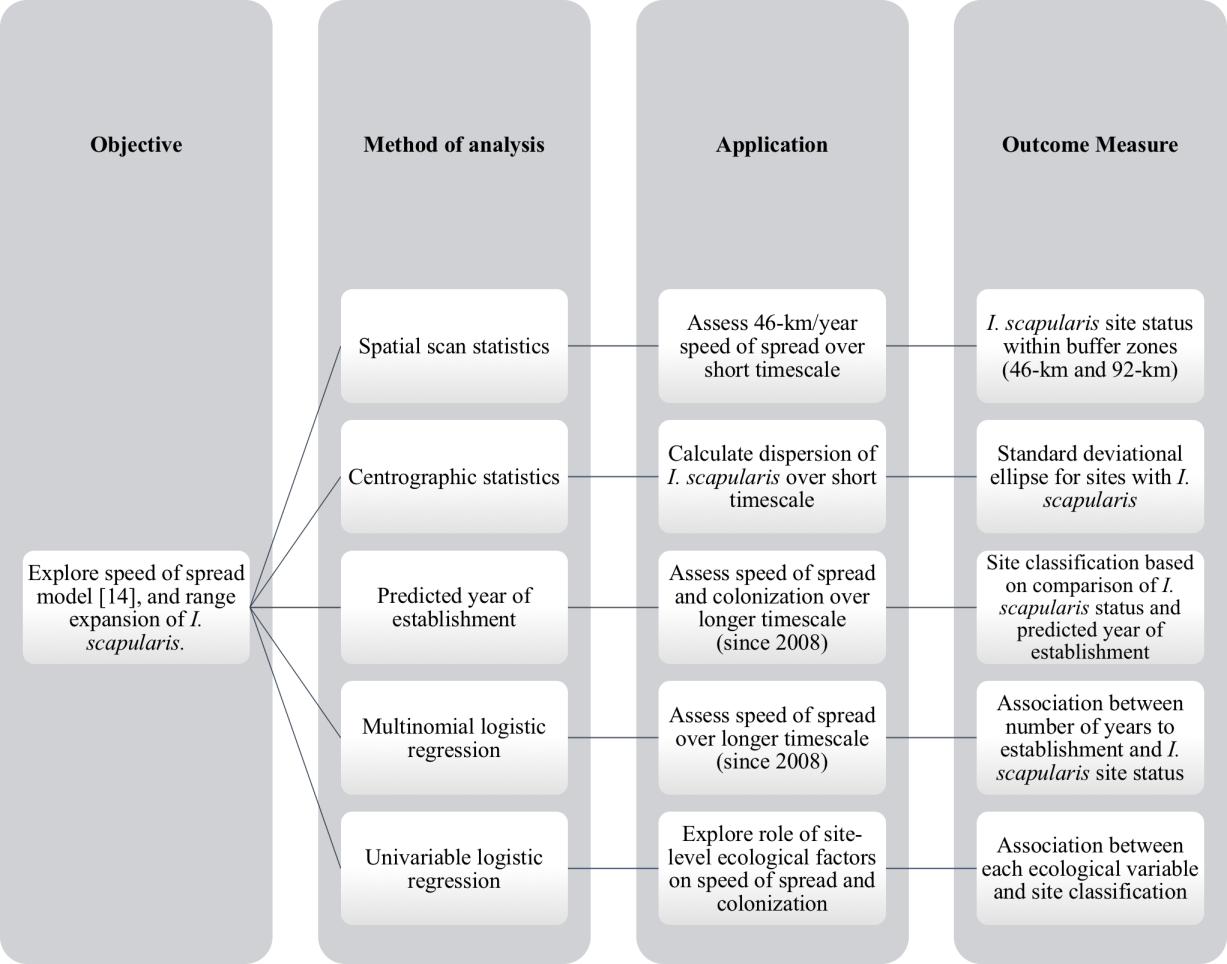
Methodology to assess speed of spread model and overall *I*. *scapularis* range expansion in Ontario. Each methodology was applied to the field data to examine a different aspect of the speed of spread model or range expansion of *I*. *scapularis*. This produced a variety of outcome measures for overall analysis and synthesis of information.

A variety of methodologies were required for model exploration, statistical analyses and spatial analyses to allow us to assess various aspects of the speed of spread model and overall range expansion of *I*. *scapularis* in Ontario (Fig 2).

## Results

### Field sampling

**Fig 3:**
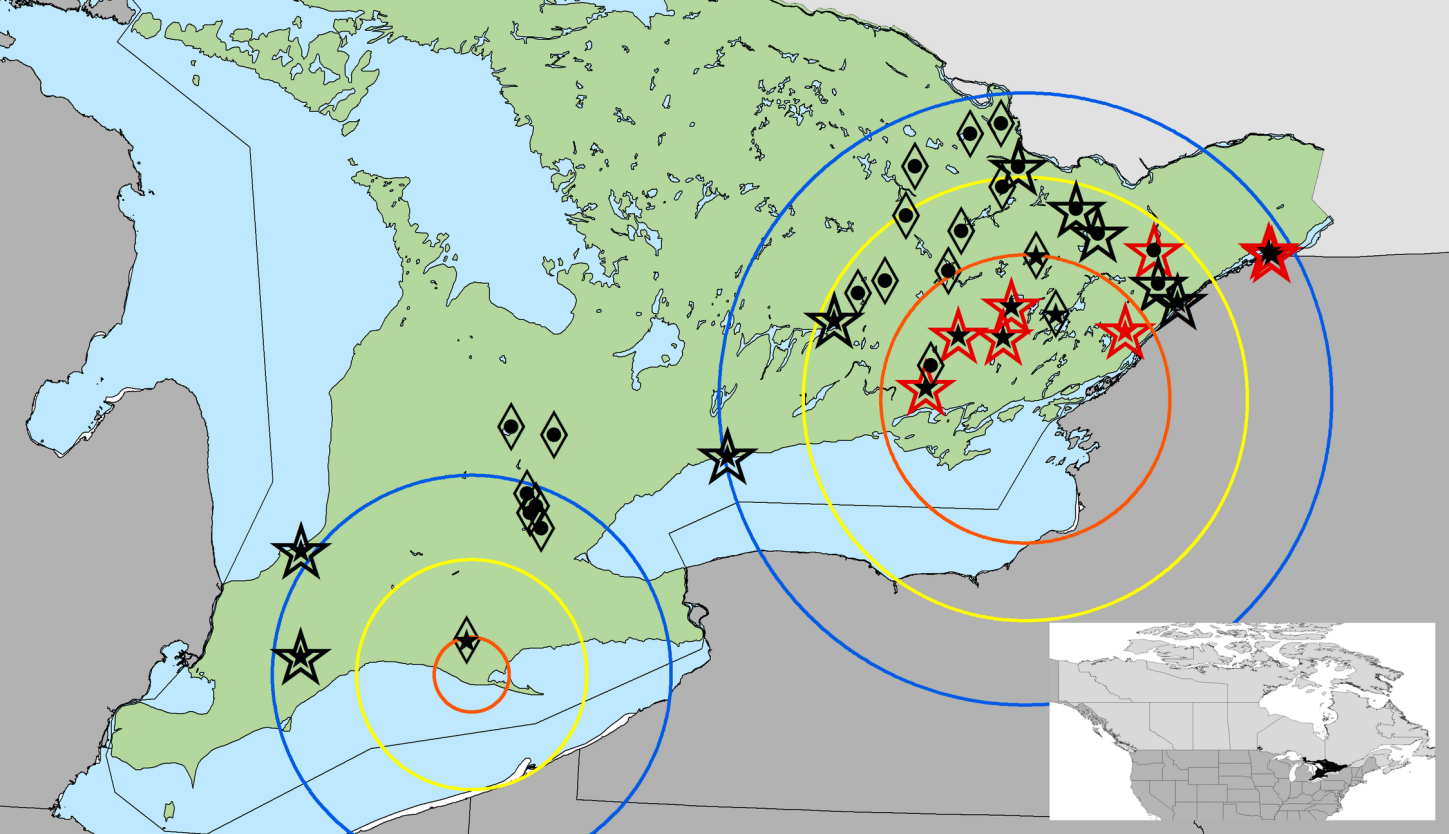
The presence of *I*. *scapularis* and *B*. *burgdorferi* at selected sites in Ontario. Thirty-six field sites were visited during the spring and fall of 2016. *Ixodes scapularis* were collected at 17 sites, of which 8 had ticks that were positive for *B*. *burgdorferi* (large hollow black stars = all ticks negative for *B*. *burgdorferi*; large hollow red stars = one or more ticks positive for *B*. *burgdorferi*); 19 were negative for the tick (large black diamond). All sites were previously visited in 2014 or 2015 (*I*. *scapularis* present, one or more ticks positive for *B*. *burgdorferi* = small red star; *I*. *scapularis* present, ticks negative for *B*. *burgdorferi* = small black stars; *I*. *scapularis* absent = small black dots). The two spatial clusters (orange lines) detected in eastern Ontario (primary cluster) and southern Ontario (secondary cluster) [8,17], and the 46 km (yellow lines) and 92 km (blue lines) buffers representing one or two years of *I*. *scapularis* spread, respectively, are provided for reference [14]. An inset map is also included.

*Ixodes scapularis* was detected at 17 of the 36 sites in 2016 (47.2%; 95% CI 30.4%-64.5%). This was the first detection of *I*. *scapularis* at five sites (Table 1; Fig 3); four of these sites were within the 46-km buffer of the primary cluster, and the remaining site was within the 92-km buffer of the primary cluster. No changes were detected around the secondary cluster.

**Table 1:** Field sampling and laboratory results from the baseline (2014–2015) and follow-up (2016) field sampling time frames.

Site status	Baseline (2014–2015) field sampling [8,17]	Follow-up (2016) field sampling
Presence of *I*. *scapularis*	29/154 sites	17/36 sites (5 newly detected) (total = 34/154)
Presence of *I*. *scapularis* that were positive for *B*. *burgdorferi*	9/29 sites	8/17 (7 newly detected) (total = 16/34)

### Laboratory analyses

Samples of *I*. *scapularis* from 16 sites were tested (number of ticks = 56; median = 2/site; range = 1-11/site), and 8 sites were positive for *B*. *burgdorferi* (50.0% (95% CI 24.6%-75.3%)). Seven of these sites were positive for *I*. *scapularis*, but negative for *B*. *burgdorferi* in 2014 or 2015. One site was negative for both the tick and bacteria at the first sampling, but positive for *I*. *scapularis* and *B*. *burgdorferi* at the second sampling. All 16 sites were negative for *A*. *phagocytophilum*, *B*. *microti*, *B*. *miyamotoi* and Powassan virus (0% (one-sided 97.5% CI 0%– 20.6%) (Table 1).

### Model exploration

For 18 sites, the sampling period occurred outside of the time frame of the prediction (i.e., predicted year of establishment pre-2014 or post-2016) (S1 Table). Eight sites were sampled after the predicted year of establishment (and confidence interval), and were classified as established. Ten sites were sampled before the predicted year of establishment; six of these sites were sampled outside of the confidence interval of the prediction. All ten sites were negative for *I*. *scapularis*. The remainder of the sites were sampled within the time frame of the prediction (S1 Table). At four sites classified as established, *I*. *scapularis* was detected prior to the predicted year of establishment. Seven sites were negative for *I*. *scapularis* after the predicted year of establishment and outside of the confidence interval for the prediction, while four sites were negative for *I*. *scapularis* after the predicted year of establishment, but within the confidence interval of the prediction. Three sites were not assessed because *I*. *scapularis* was detected at the first visit, but not at the follow-up visit.

The average DD>0°C for the time frame preceding the study was higher than the average DD>0°C from 1990–2008 for all CSDs (mean 149.53; range 53.35–216.04). The estimated speed of range expansion therefore could to be greater than 46 km/year, but not exceed 55 km/year.

### Statistical analyses

The odds of a site being established for *I*. *scapularis* or a risk area for *I*. *scapularis* when compared to sites that were absent for *I*. *scapularis* significantly decreased as the number of years to establishment increased, based on multinomial logistic regression (Table 2). The assumption of linearity was met, and the model adequately fit the data.

**Table 2:** The association between the number of years to establishment from 1991 and the *I*. *scapularis* site classification based on multinomial logistic regression.

Outcome Levels	Explanatory variable	Relative risk ratio (95% confidence interval)	p-value
Level 1 (base level) = Absent	Years to establishment		
Level 2 = Risk area		0.60 (0.40–0.89)	0.010
Level 3 = Established		0.62 (0.44–0.87)	0.006

No ecological variables were significant for sites late-to-establish for *I*. *scapularis* based univariable logistic or exact logistic regression (p>0.20) (S1 Table).

### Spatial analyses

**Fig 4:**
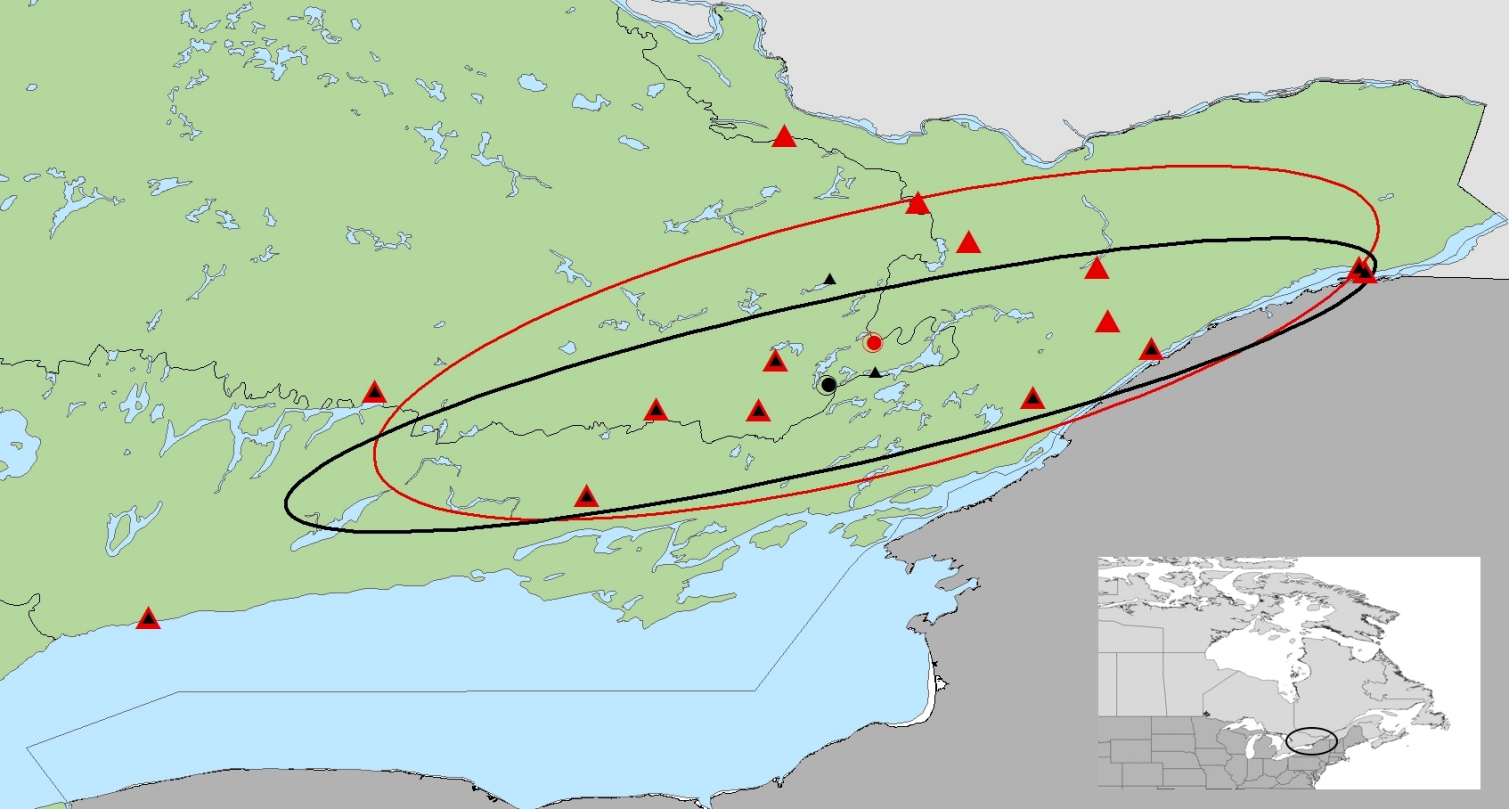
Dispersion of sites positive for *I*. *scapularis* for each field sampling time frame. Baseline field sampling in 2014 and 2015 detected 12 sites with *I*. *scapularis* in eastern Ontario (site = black dot, mean center = black triangle). Repeat follow-up field sampling in 2016 detected *I*. *scapularis* at 15 sites (site = red dot, mean center = red triangle). The dispersion of sites with *I*. *scapularis* increased within this area, based on the standard deviational ellipse (baseline = black ellipse, follow-up = red ellipse). An inset map is provided for reference.

The dispersion of sites positive for *I*. *scapularis* within the area of the primary cluster and buffers, as represented by a standard deviational ellipse, increased between the baseline field sampling season (2014–2015) and the follow-up field sampling season (Table 3; Fig 4.). The area of the standard deviational ellipse increased by 6391.51 km^2^ northward over the two sampling time frames (Fig 4). Although no calculations could be conducted for the secondary cluster, no changes were noted in *I*. *scapularis* site status in this area between the field sampling time frames.

**Table 3:** Centrographic statistics of the primary cluster and buffer regions for the baseline (2014–2015) and follow-up (2016) field sampling seasons.

Centrographic statistic	Primary cluster and buffer region	
	Baseline (2014–2015) (n = 12)	Follow-up (2016) (n = 15)
**Mean centre x** (standard deviation)	-76.556008 (1.212581)	-76.403454 (1.127639)
**Mean centre y** (standard deviation)	44.641405 (0.327147)	44.781788 (0.398138)
**Standard Deviational Ellipse**		
Clockwise angle of rotation	72.10^o^	69.79^o^
SD of x axis	29.77 km	46.83 km
SD of y axis	149.27 km	138.36 km
Area	13962.89 km^2^	20354.40 km^2^

## Discussion

Range expansion of *I*. *scapularis* into Canada has been documented since the early 1990s and is predicted to continue northward, in part due to climate change [10,14,20]. The emergence of *I*. *scapularis*-borne pathogens, most notably *B*. *burgdorferi*, has occurred following the introduction of the tick. Previous field sampling has demonstrated that *I*. *scapularis*, including those carrying *B*. *burgdorferi*, can be found in southern, eastern and central Ontario, with a hot spot in eastern Ontario [8,17]. In this study, we have illustrated the ongoing invasion of *I*. *scapularis*, and *B*. *burgdorferi* within Ontario. With this data, we have examined the processes associated with *I*. *scapularis* spread over a short timescale, including the speed of range front expansion estimated by Leighton et al. [14], and *I*. *scapularis* colonization into suitable woodland behind the range front.

Follow-up field sampling from Clow et al. [8,17] detected *I*. *scapularis* at 17 out of 36 sites. Five of these sites were newly documented sites for the tick, and located within either the 46-km buffer (4 sites) or the 92 km-buffer (1 site) of the primary cluster. The dispersion of positive sites in eastern Ontario increased in a northward direction between sampling time frames. This area may represent the range front of *I*. *scapularis* expansion in eastern Ontario, and based on a short timescale, provide data to support the speed of spread estimate of 46 km/year. However, when these sites are compared with the model prediction for year of establishment of *I*. *scapularis*, all five sites were late-to-establish. If these sites represent the range front of expansion of *I*. *scapularis*, then the process may be occurring at a slower rate than estimated, especially since recent temperature conditions were elevated, or be occurring at an inconsistent rate that is not detectable over the short timescale.

Site status was compared with the predicted year to establishment for each CSD for the remaining sites. For 18 sites, our sampling time frame was outside of the predicted year of establishment, so it was not possible to establish a temporal relationship. These sites were however in general alignment with the predictions (i.e., post-prediction and established or pre-prediction and absent). For the sites that we could establish a temporal relationship, 4 were early to establish, and 6 were late-to-establish, when compared to the prediction. These findings illustrate that behind the range front, *I*. *scapularis* colonization is occurring heterogeneously, and does not consistently align with the same timescale of range front expansion. That being said, there still appears to be a relationship with the predicted year of establishment from the model and field data illustrating the colonization of *I*. *scapularis*. Based on the multinomial logistic regression, the predicted number of years to establishment was a significant explanatory variable for site status; the odds of a site being classified as a risk area or as an established area, in comparison to the base level of a negative site, decreased with an increasing number of predicted years to establishment from the speed of spread model.

Both processes of range front expansion and colonization or ‘filling-in’ of woodland habitats behind the range front are dependent on suitable ecological conditions. The speed of spread model incorporates the ecological factors of cumulative degree days above zero (^o^C), precipitation, elevation, and long-distance and local dispersal at the broad-level of census sub-division [14]. These factors most likely influence both processes, and explain the general predictive ability of the model for both processes associated with *I*. *scapularis* spread.

We know that other ecological factors, such as the density and composition of the understory, the depth of litter layer and the type of forest create favourable habitat for *I*. *scapularis* [17, 28–31]. Examination of these factors at a finer spatial scale may be needed to understand the colonization process as the microclimate and microhabitat have a significant impact on the tick [32]. To explore what additional ecological factors may influence this colonization process, we conducted univariable analysis using logistic regression. The outcome was the late-to-establish site classification (in comparison to all other site classifications), and the explanatory variables were site aspect, forest type, understory density and type, soil composition and moisture level, the depth of the litter layer, and the difference in DD>0°C between 2009–2013 and 1990–2008. Based on this analysis, there is no evidence that these ecological factors have contributed to the heterogeneous process of *I*. *scapularis* colonization. It is important to note that our sample size was small (n = 33) and therefore our power to detect a difference was limited.

Consideration also needs to be given to the host population. The role of migratory birds in long distance dispersal (≤425 km) is highly likely, and included in the speed of spread model [9]. Deer and small mammals are important hosts for the adult and immature life stages, respectively, and have been positively associated with tick abundance in Ontario [29,31]. The role these species play in dispersal on a local level is less well understood. Madhav and colleagues [13] developed a model that incorporated home range, tick burden and density of white-tailed deer, white-footed mice and American Robins to illustrate how *I*. *scapularis* would disperse over a simple landscape. Deer had the greatest role in dispersal because of their comparatively large home range (~5 km) and large burden of gravid females. White-footed mice showed a negative association with range expansion due to their small home ranges and large burden of immature ticks [13]. It is unknown if this model is applicable to more complex multi-host systems in nature, as there has been limited field research in this area. Further investigation into the host dynamics on a local scale may provide additional insight into the process of *I*. *scapularis* spread, and allow us to refine our predictions for future spread for both range front expansion and colonization.

Of particular interest would be further assessment of habitat and host factors between the areas surrounding the primary and secondary clusters. Based on our field sampling, spread of *I*. *scapularis* was only detected in eastern Ontario. We suspect that the vast areas of agricultural land, as well as densely populated urban areas in southern Ontario may impact the speed of *I*. *scapularis* spread. In contrast, eastern Ontario has greater woodland habitat and less developed area, which may facilitate *I*. *scapularis* range expansion [33,34].

It is important to note that surveillance approaches have evolved since the risk of *I*. *scapularis* was first identified. The passive surveillance program in Ontario has been in place for decades [35]. However, passive surveillance can be highly influenced by the level of effort placed into the recruitment of samples. Programs such as ‘Let’s Target Lyme’, led by Public Health Ontario in 2010, have contributed to increased public awareness and sample submission [34]. Therefore, the passive surveillance data on which the speed of spread model is founded may have varied in quality and quantity from 1991–2008 and thus impacted the development of the model [14].

Emergence of *B*. *burgdorferi* in ticks was detected at a subset of sites where *I*. *scapularis* had been previously documented (either in 2014 or 2015). The tick and the pathogen were collected for the first time concurrently at one site. Three nonexclusive hypotheses exist for the emergence of *B*. *burgdorferi*: tick-first, pathogen-first or dual invasion [36]. For the tick-first hypothesis, *I*. *scapularis* is introduced via long distance dispersal on hosts and do not initially establish a cycle of *B*. *burgdorferi* transmission. This can occur if the primary hosts for dispersal are white-tailed deer and they introduce fed, uninfected adult ticks (since deer are not competent reservoirs, and can clear spirochete infection from ticks) [36]. This can also occur if fed nymphal ticks (infected or uninfected) are introduced into an area, and then feed on white-tailed deer as adults, resulting in no further sustained transmission of *B*. *burgdorferi* [16]. The pathogen may be introduced later via infected ticks or hosts. The pathogen first hypothesis explores the potential of alternate host and tick species maintaining *B*. *burgdorferi* in a cryptic cycle without *I*. *scapularis*. Risk of pathogen transmission to humans can develop rapidly if *I*. *scapularis* is later brought into the area. When the tick and pathogen are introduced concurrently via mammalian and avian hosts, dual invasion has occurred [36].

Which process(es) occurs depends on a variety of ecological factors, including long and local dispersal patterns, host abundance, tick phenology, and habitat suitability. Over five years of field sampling in Michigan, Hamer and colleagues [36] detected evidence of all three processes. In Canada, analysis of passive surveillance data supports the tick-first hypothesis, with the introduction of fed *I*. *scapularis* nymphs via migratory birds [8]. There is an approximate lag in *B*. *burgdorferi* transmission of five-years in eastern Canada [16]. As we only had two site visits over a period of three years and collected a small number of *I*. *scapularis* (total = 56 adult and nymphal ticks, median = 2/site), we are not able to establish a temporal relationship with the emergence of *I*. *scapularis* and the transmission of *B*. *burgdorferi*. We also did not take blood samples from hosts for serology or collect any other tick species via dragging, so rigorous assessment of the pathogen-first hypothesis was not conducted. In general, our findings are consistent the tick-first hypothesis, as most sites where *I*. *scapularis* recently emerged had ticks that were negative for *B*. *burgdorferi*, and ticks at several sites remained negative for *B*. *burgdorferi* over two field sampling seasons. It is necessary to continue to collect field data, including alternate tick species and serological samples from hosts, in and around eastern Ontario over a longer time frame to assess the three hypotheses and validate the prediction for the speed of *B*. *burgdorferi* emergence.

There are several limitations of this study that need to be acknowledged. First, we had a small sample size and sampling time frame. It would have been ideal to revisit all field sites for more than one follow-up field season. However, this was not feasible for this study, and as a result, we have lower power for statistical analyses (Type II error) and reduced ability to illustrate invasion over time. Also, there are some limitations associated with our method for *I*. *scapularis* collection. Tick dragging can have low sensitivity, especially in areas of emergence where the tick density is low, and can be highly variable, depending on the time of day and weather conditions [7,37]. We expect that some of our sites are falsely negative, and therefore may not be late-to-establish.

Lyme disease has been recognized as an emerging vector-borne disease of public health significance in Canada [38]. Our study provides valuable information for public health interventions in Ontario. To appropriately target surveillance efforts, public education and physician and veterinarian awareness, it is crucial to know the current distribution of *I*. *scapularis* and *B*. *burgdorferi*. We previously established a baseline distribution, and with our follow-up field sampling, have highlighted new areas of risk. Since these changes were documented over a short period of time, our findings also emphasize the need for the public health, medical and veterinary medical professionals to remain vigilant and aware of the changing risk of *I*. *scapularis*-borne disease.

In the future, efforts should be placed into ongoing field sampling, especially around eastern Ontario, which is a hot spot for the tick. Additional research should be considered to understand the role that the local host population plays in the dispersal of *I*. *scapularis*.

## Conclusions

We have illustrated the ongoing spatial spread of *I*. *scapularis* and the emergence of *B*. *burgdorferi* in Ontario, Canada, especially around the hot spot in eastern Ontario. With field collected data, we explored the speed of spread model for *I*. *scapularis* and showed that on a short timescale, the process of *I*. *scapularis* invasion is consistent with the estimated rate of 46 km/year. However, this speed may not be uniform and the following colonization of *I*. *scapularis* behind the range front is occurring at a heterogeneous rate. These findings can be used by public health officials to target preventative interventions. In the future, ongoing field sampling is needed to validate the model for the speed of *B*. *burgdorferi* invasion, as well as to understand the role host species may play in local dispersal of the tick.

## Supporting information

S1 Table Comparison of the *I*. *scapularis* site status over two years of field sampling with the predicted year to establishment based on Leighton et al. (2012) at 33 sites in Ontario. (DOCX)

S2 Table The univariable analysis of site-level ecological variables on the late establishment of *I*. *scapularis* at 33 sites sampled in Ontario during the spring, summer and fall of 2014 or 2015 and again in 2016 based on logistic regression or exact logistic regression (*). (DOCX)
