# Supplementary material for: Northward range expansion of Ixodes scapularis evident over a short timescale in Ontario, Canada
Source: PLoS One. 2017 Dec 27;12(12):e0189393. doi: 10.1371/journal.pone.0189393 (PMC5744917; doi:10.1371/journal.pone.0189393)
Supplement: S1 Table — (DOCX) [file pone.0189393.s001.docx]

**S1 Table 1: Comparison of the *I. scapularis* site status over two years of field sampling with the predicted year to establishment based on Leighton et al. (2012) at 33 sites in Ontario.**

| **Site ID** | ***I. scapularis* status of initial visit in 2014** | ***I. scapularis* status of initial visit in 2015** | ***I. scapularis* status at follow-up visit in 2016** | ***I. scapularis* site classification** | **Predicted year of establishment** | **Confidence Interval** | **Assessment of Prediction** |
| --- | --- | --- | --- | --- | --- | --- | --- |
| 1 | Yes | - | Yes | Established | 2006 | 2006-2007 | Post-prediction |
| 2 | Yes | - | Yes | Established | 2015 | 2013-2017 | Early |
| 4 | Yes | - | Yes | Established | 2015 | 2013-2017 | Early |
| 5 | Yes | - | Yes | Established | 2010 | 2009-2010 | Post-prediction |
| 13 | No | - | No | Negative | 2023 | 2019-2027 | Pre-prediction |
| 21 | No | - | Yes | Risk area | 2010 | 2009-2012 | Late |
| 22 | No | - | Yes | Risk area | 2012 | 2011-2013 | Late |
| 24 | No | - | Yes | Risk area | 2008 | 2008-2009 | Late |
| 25 | No | - | Yes | Risk area | 2007 | 2006-2007 | Late |
| 26 | Yes | - | Yes | Established | 2007 | 2006-2008 | Post-prediction |
| 48 | No | - | No | Negative | 2024 | 2020-2029 | Pre-prediction |
| 66 | Yes | - | Yes | Established | 2011 | 2010-2012 | Post-prediction |
| 67 | Yes | - | Yes | Established | 2011 | 2010-2012 | Post-prediction |
| 69 | No | - | No | Negative | 2014 | 2013-2016 | Late |
| 88 | No | - | No | Negative | 2012 | 2011-2013 | Late |
| 92 | Yes | - | Yes | Established | 2005 | 2005-2006 | Post-prediction |
| 95 | Yes | - | Yes | Established | 2005 | 2005-2006 | Post-prediction |
| 101 | No | - | No | Negative | 2014 | 2012-2016 | Late |
| 115 | - | No | No | Negative | 2021 | 2017-2025 | Pre-prediction |
| 116 | - | No | No | Negative | 2023 | 2019-2029 | Pre-prediction |
| 117 | - | No | No | Negative | 2021 | 2017-2025 | Pre-prediction |
| 119 | - | No | No | Negative | 2026 | 2021-2031 | Pre-prediction |
| 120 | - | Yes | Yes | Established | 2017 | 2014-2020 | Early |
| 122 | - | Yes | Yes | Established | 2019 | 2016-2021 | Early |
| 128 | - | Yes | Yes | Established | 2010 | 2009-2010 | Post-prediction |
| 129 | - | No | No | Negative | 2018 | 2015-2021 | Pre-prediction |
| 139 | - | No | Yes | Risk area | 2015 | 2013-2017 | Late |
| 149 | - | No | No | Negative | 2017 | 2015-2019 | Pre-prediction |
| 150 | - | No | No | Negative | 2016 | 2014-2018 | Late |
| 151 | - | No | No | Negative | 2015 | 2013-2017 | Late |
| 152 | - | No | No | Negative | 2015 | 2013-2017 | Late |
| 153 | - | No | No | Negative | 2019 | 2016-2022 | Pre-prediction |
| 154 | - | No | No | Negative | 2019 | 2016-2022 | Pre-prediction |
