# Supplementary material for: Northward range expansion of Ixodes scapularis evident over a short timescale in Ontario, Canada
Source: PLoS One. 2017 Dec 27;12(12):e0189393. doi: 10.1371/journal.pone.0189393 (PMC5744917; doi:10.1371/journal.pone.0189393)
Supplement: S2 Table — (DOCX) [file pone.0189393.s002.docx]

**SI Table 2: The univariable analysis of site-level ecological variables on the late establishment of *I. scapularis* at 33 sites sampled in Ontario during the spring, summer and fall of 2014 or 2015 and again in 2016 based on logistic regression or exact logistic regression (*).**

| **Explanatory variable** | **Category**  **(number of observations)** | **Odds ratio (95% confidence interval); p-value** |
| --- | --- | --- |
| Aspect category* | Flat (19)  Incline (2)  Variable (12) | REF  4.36^[[1]](#footnote-1)^(0.32- +Inf.); 0.267  0.73 (0.09-4.61); 1.000 |
| Forest type* | Coniferous (6)  Deciduous (15)  Mixed (12) | REF  0.68 (0.07-6.91); 1.000  0.22(0.012-2.90); 0.352 |
| Understory density* | Sparse (11)  Full (16)  Dense (2) | REF  0.29 (0.04-1.86); 0.248  0.85 (0.01-78.36); 1.000 |
| Predominant understory type* | Mixed (16)  Non-woody (7)  Trees (6) | REF  0.29 (0.01-3.46); 0.551  3.15 (0.33-45.11); 0.458 |
| Predominant soil type* | Mixed (8)  Clay (6)  Loam (14)  Sand (5) | REF  0.36 (0.53-6.48); 1.000  0.68 (0.08-6.53); 1.000  2.32 (0.16-44.95); 0.8252 |
| Soil moisture* | Dry (3)  Fresh (17)  Moist (12)  Wet (1) | REF  1.38 (0.06-94.23); 1.000  0.43 (0.01-35.28); 1.000  1.0*^[[2]](#footnote-2)^ (0.03- +Inf); 1.000 |
| Depth of litter layer (cm) | Continuous variable (33) | 1.10 (0.62-1.96); 0.733 |
| Loam soil (%) | Continuous variable (33) | 1.01 (0.98-1.04); 0.667 |
| Clay soil (%) | Continuous variable (33) | 0.984 (0.95-1.01); 0.291 |
| Sand soil (%) | Continuous variable (33) | 1.01 (0.98-1.05); 0.453 |
| Difference in average Cumulative DD>0^O^C  (Average DD>0^O^C 2009-2013 - Average 1991 to 2008) | Continuous variable (33) | 1.00 (0.958-1.02); 0.623 |

1. Median unbiased estimate [↑](#footnote-ref-1)
2. Median unbiased estimate [↑](#footnote-ref-2)
